# Postpartum Depression Is Associated With Altered Neural Connectivity Between Affective and Mentalizing Regions During Mother-Infant Interactions

**DOI:** 10.3389/fgwh.2021.744649

**Published:** 2021-09-23

**Authors:** Judith K. Morgan, Hendrik Santosa, Rachel M. Fridley, Kaetlyn K. Conner, Alison E. Hipwell, Erika E. Forbes, Theodore J. Huppert

**Affiliations:** ^1^Department of Psychiatry, University of Pittsburgh, Pittsburgh, PA, United States; ^2^Department of Psychology, University of Pittsburgh, Pittsburgh, PA, United States; ^3^Department of Radiology, University of Pittsburgh, Pittsburgh, PA, United States; ^4^University of Pittsburgh Medical Center, Pittsburgh, PA, United States; ^5^Department of Electrical and Computer Engineering, University of Pittsburgh, Pittsburgh, PA, United States

**Keywords:** postpartum depression, mentalizing, maternal sensitivity, near infrared spectroscopy, neural connectivity, maternal brain network

## Abstract

Although there has been growing interest in mood-related neural alterations in women in the initial weeks postpartum, recent work has demonstrated that postpartum depression often lingers for months or years following birth. However, research evaluating the impact of depression on maternal brain function during mother-infant interactions in the late postpartum period is lacking. The current study tested the hypothesis that depressive symptoms at 12-months postpartum are associated with neural alterations in affective and social neural regions, using near-infrared spectroscopy during *in vivo* mother-infant interactions. Participants were 23 birth mothers of 12-month-old infants (60% boys). While undergoing near-infrared spectroscopy, mothers engaged in an ecologically valid interactive task in which they looked at an age-appropriate book with their infants. Mothers also reported on their depressive symptoms in the past week and were rated on their observed levels of maternal sensitivity during mother-infant play. Greater depressive severity at 12-months postpartum was related to *lower* connectivity between the right temporoparietal junction and the lateral prefrontal cortex, but *greater* connectivity between the right temporoparietal junction and anterior medial prefrontal cortex during mother-infant interaction. Given the putative functions of these neural regions within the maternal brain network, our findings suggest that in the context of depression, postpartum mothers' mentalizing about her infants' thoughts and feelings may be related to *lower* ability to express and regulate her own emotions, but *greater* ability to engage in emotional bonding with her infant. Future work should explore how connectivity among these regions is associated with longitudinal changes in maternal behavior, especially in the context of changes in mothers' depressive symptoms (e.g., with treatment) over time.

## Introduction

At least one in seven new mothers are affected annually with postpartum depression (1). Postpartum depression (PPD) is characterized by low mood and feelings of inadequacy, guilt, and despair and often onsets by 6–12 weeks postpartum (2). Research has also shown that PPD often lingers for months or years following birth (3). One study demonstrated that more than half of women who had been diagnosed with a psychiatric disorder in the first 2 months postpartum were still symptomatic in the second half of the postpartum year (4). Beeghly et al. (5) found that 31% of women who had elevated depressive symptoms at 2 months postpartum continued to report elevated depressive symptoms at 12 months postpartum. Some conceptual models suggest that PPD that persists past the early postpartum period (vs. resolving in the early weeks or months) is often characterized by factors such as lower socioeconomic status, poor social support, and greater depression severity (3).

This emerging evidence that PPD often persists for months or years is troubling as symptoms of PPD, particularly loss of interest or pleasure, can result in detachment or disengagement from social interactions, thereby interfering with the provision of warm, sensitive caregiving (6). Indeed, prior research suggests that depressed mothers may be poorly attuned to infant cues, perhaps due to depression-related disruptions in interpersonal functioning (7–9). A greater understanding of how socio-affective neural processes, such as those that support providing sensitive care to one's infant, may be negatively impacted by PPD in the late postpartum period could be useful in understanding caregiving disruptions that persist over time and influence infant socio-emotional development.

Some models describe the postpartum period as a time of increased “caring concern,” the combination of extreme concern over the wellbeing of one's infant and worry or doubts in one's ability to provide optimal care (10, 11). Mothers with postpartum psychiatric illness, including PPD, may experience heightened levels of caring concern (12). Indeed, theoretical models and empirical research suggest that depression is a disorder in which empathic concern for others is paired with misattributions of self-blame, leading to heightened feelings of guilt and responsibility for having caused distress in others (13–15). In this regard, for some mothers with PPD, increased caring concern may be indicative of *greater* effort to provide sensitive care for her infant, perhaps to avoid or diminish disproportionate feelings of guilt for her infant's distress. This may translate to mothers who allocate extra resources to sensitively attune to her infant's cues despite her own levels of dysphoria and/or dysregulated affect. Thus, there may be some neural profiles [e.g., see (16)] that are indicative of sensitive caregiving in the midst of postpartum depression.

More specifically, neural regions implicated in social cognition, such as mentalizing or the ability to understand the thoughts and feelings of others (e.g., temporoparietal junction), and in social bonds (e.g., anterior medial prefrontal cortex) appear to underlie healthy interpersonal functioning, including positive interactions with one's own baby (17–20). Medial and lateral prefrontal regions have been demonstrated to play a role in affective processing, including in the expression and regulation of emotions (21, 22). The anterior medial prefrontal cortex has been implicated in affectionate touch and mother-infant bonding (23). Other work has demonstrated that the right temporoparietal junction (TPJ) appears to aid in mentalizing and in orienting to salient information in the environment (24, 25), which are also important components for maternal care. Morgan et al. (16) also found that greater activity in the TPJ in postpartum mothers was associated with greater sensitivity in caregiving.

Indeed, the medial and lateral prefrontal cortices and the TPJ, among other regions, have been identified as part of a network of brain regions deemed the “maternal brain network” (18). A growing body of work that has compared parents to non-parents and/or evaluated neural response to personally relevant parenting stimuli (e.g., own baby's cry) has shown significant neural differences in the maternal brain network that have been attributed to the transition to parenthood [for a review, see (20)]. As noted above, healthy function in the maternal brain network serves to facilitate the provision of sensitive care, likely because these regions aid in helping parents (1) regulate their own emotions *via* affective processing regions (e.g., lateral prefrontal cortex), (3) bond with their babies *via* affectionate touch and emotional expression (e.g., anterior medial prefrontal cortex), and (2) sensitively attune to their infants' cues *via* the capacity for mentalizing (e.g., TPJ). Most notably, the maternal brain network may be altered in the context of PPD, thereby also interfering with the provision of sensitive caregiving (18). Similar to broader research showing lower responding in affective and social processing regions (e.g., prefrontal cortex and TPJ) in clinically depressed adults, women with PPD also show diminished activity in these regions (26, 27). Regions within the maternal brain network must work in tandem to effectively promote regulated maternal emotion and sensitive maternal behavior. In healthy postpartum mothers, *greater* activity in mentalizing regions in response to infant cues is likely associated with *greater* activity in regions that promote expression of affectionate touch and bonding (e.g., anterior medial regions) and *greater* activity in regions that aid in emotional self-regulation. However, less is known about how these regions work together in postpartum mothers coping with depressive symptoms.

Near infrared spectroscopy (NIRS) is an especially advantageous technique for understanding the functional neural correlates of maternal behavior. NIRS is non-restrictive and less sensitive to movement than fMRI or EEG, allowing for assessment of brain activity while mothers interact naturalistically with their babies (28), providing the opportunity to measure brain activity in ecologically valid contexts for human maternal behavior. Additionally, most research on neural mechanisms of PPD, including studies using NIRS, has relied on standardized, computer-based paradigms [e.g., video or audio stimuli, for a review see (29)] rather than assessing brain activity during naturalistic mother-infant play. Evaluating neural activity during naturalistic mother-infant play could better elucidate the ways in which neural connectivity in the maternal brain network may be altered in the context of postpartum depression, especially during the late postpartum period in which the infant has a directive role in the interaction.

In summary, there is a lack of research evaluating the impact of depression on maternal brain function in the late postpartum period using ecologically valid methods. The current study evaluated whether postpartum depressive symptoms present at 12-months postpartum are related to neural alterations in affective and social neural regions, using near-infrared spectroscopy during ecologically valid, *in vivo* mother-infant interactions. We hypothesized that greater severity of postpartum depressive symptoms would be associated with lower connectivity between the temporoparietal junction and bilateral medial and lateral prefrontal cortex in mothers when interacting with their 12-month-old infants. We explored whether depression-related alterations in neural connectivity would be related to the provision of sensitive care during the late postpartum period.

## Methods

Participants were 23 birth mothers (*M*age = 31.41 years, SD = 3.57, *Range* = 23–38 years) of 12-month-old infants (61% boys, *M*age = 12.04 months, *SD* = 1.40 months, *Range* = 10–14-months). Mother-infant dyads were recruited from the community using a University sponsored research registry. Mothers were eligible for the study if they were currently experiencing elevated depressive symptoms *or* had no prior lifetime history of any psychiatric illness. Mothers were required to be free of bipolar disorder or schizophrenia and infants were required to be free of developmental delays or disabilities or serious medical conditions.

Of the 23 participating mothers, 65% identified as White, 17% as Black/African American, and 9% as Asian. One mother identified as Latinx. Further, 78% indicated that they were married (*n* = 17) or living with a partner (*n* = 1) and 21% indicated that they were single (*n* = 3) or separated (*n* = 1). The majority of mothers reported having some type of post-secondary education (*n* = 19). Nine mothers reported being first time mothers. Originally, one additional mother was assessed but her brain data were not included due to technical difficulties with the NIRS equipment.

### Procedure

Mothers were interviewed on their current and prior history of depression and other psychiatric illnesses. On a subsequent visit (*M* = 35.87 days, *SD* = 30.06 days), mothers and infants engaged in a free play activity with a set of standard developmentally appropriate toys and this interaction was videotaped for later coding of maternal sensitivity. Next, while undergoing NIRS, mothers engaged in an ecologically valid interactive task in which they looked at an age-appropriate book with their infants for 3 min. At the time of the mother-infant play visit, mothers rated their depressive symptoms in the past week using the Center for Epidemiological Studies-Depression (CES-D). This research was approved by the University of Pittsburgh Human Research Protections Office and all mothers provided informed consent before participation in the study.

### Measures

#### Depression

Mothers were interviewed on their depressive symptoms and other psychiatric illnesses using the Structured Clinical Interview for DSM-IV (SCID-IV) (30), a semi-structured clinical interview designed to assess lifetime psychiatric history, by Bachelors' and Masters' level trained interviewers. Seventeen percent of interviews (*n* = 4) were double coded by a licensed clinical psychologist to ensure reliability (95% agreement). At the time of the NIRS assessment and mother-infant interaction, mothers rated severity of depressive symptoms in the past week on a 4-point Likert scale with 0 = rarely or none of the time (<1 day) and 3 = most or all the time (5–7 days). Sample items on the CES-D include “I felt depressed” and “I felt that I could not shake off the blues even with the help from my family and friends.” A score of 16 or higher on the CES-D has been identified as a clinical cutoff for likely depression (31). Internal consistency was high for the CES-D in this sample (α = 0.95).

Eleven mothers were characterized as depressed because they met current criteria for Major Depressive Disorder or Depressive Disorder NOS on the SCID (*n* = 8) and/or had elevated scores on the Center for Epidemiological Studies-Depression (CES-D) scale (≥16, *n* = 8). Seven of these 11 mothers also met current or lifetime criteria for one or more anxiety or trauma-related disorders (*n* = 4 Generalized Anxiety Disorder, *n* = 4 Post-Traumatic Stress Disorder, *n* = 2 Panic Disorder, *n* = 1 Social Anxiety Disorder, *n* = 1 Specific Phobia, *n* = 1 Obsessive Compulsive Disorder). Of these 11 mothers, 4 were currently taking an antidepressant medication (i.e., Venlafaxine, Escitalopram, or Sertraline). The remaining 12 mothers were healthy in the postpartum period, did not meet current or lifetime criteria for any psychiatric illness on the SCID, and had a score lower than 16 on the CES-D. Due to limited sample size, we used the CES-D score as a continuous measure of depression severity in statistical models.

#### Mother-Infant Play

Mothers and infants engaged in a 10-min free play task with a standard set of toys. These included two baby dolls, a baby bottle, rattle and two baby blankets, two cars, a tool kit, a stuffed puppy, dog food and bowl, and a tea set. This task served an open-ended play task for later coding of maternal sensitivity.

Subsequently, mothers were seated at a nearby table and asked to look at an age-appropriate book with her infant, who was seated in her lap, for 3 min. All mothers and infants were given the same book, a lift the flap version of *Brown Bear, Brown Bear, What Do You See by Eric Carle*, which was chosen based on its rhyming language, colorful pictures, and flaps that allow for mother-infant interaction. As a validity check, mothers were asked to report on the frequency of looking at books with their infant at home. All mothers reported daily use and the average amount of time reported was 21.36 min (*SD* = 12.17).

For both tasks, mothers were told to play and interact with her infant as she normally would at home. Mothers' brain activity was assessed *via* NIRS during the 3-min book interaction, as it allowed for more controlled and standardized assessment of brain activity during a naturalistic interaction that has minimal movement (i.e., relative to free play) and is a proxy for mother-infant dyadic affective quality (32).

#### Maternal Sensitivity

Independent observers naïve to mother psychiatric status coded the mother-infant free play task for maternal sensitivity with the widely used Coding Interactive Behavior (CIB) (33) coding scheme. This global coding scheme has strong psychometric properties and is sensitive to sociocultural factors (34, 35) and consists of 45 codes rated on 5-point Likert scales (1 = a little, 5 = a lot). Consistent with prior postpartum research using the CIB (36), maternal sensitivity included the average of the following 10 codes: maternal acknowledgment of infant cues, clear vocal quality, positive affect, gaze, affectionate touch, appropriate range of affect, consistency of style, resourcefulness, adaptation to infant cues, and a warm, supportive presence (α = 0.79) Coders were trained to 85% reliability by a certified CIB trainer. Five videos (22%) were then double coded with the certified master coder and reliability was high for each of the 8 dimensions of the maternal sensitivity scale (ICCs = 0.86–0.98).

#### Sociodemographic Risk

We calculated a sociodemographic risk variable by dummy coding responses for maternal education (0 = some college education or higher, 1 = high school education or lower) and maternal relationship status (0 = married or living with a partner, 1 = single or separated). Mothers were coded as 1 for the Sociodemographic Risk Factor if they had a score of 1 on either maternal education or relationship status and were coded as 0 if they had no risk factors.

### fNIRS Data Collection

**Figure 1:**
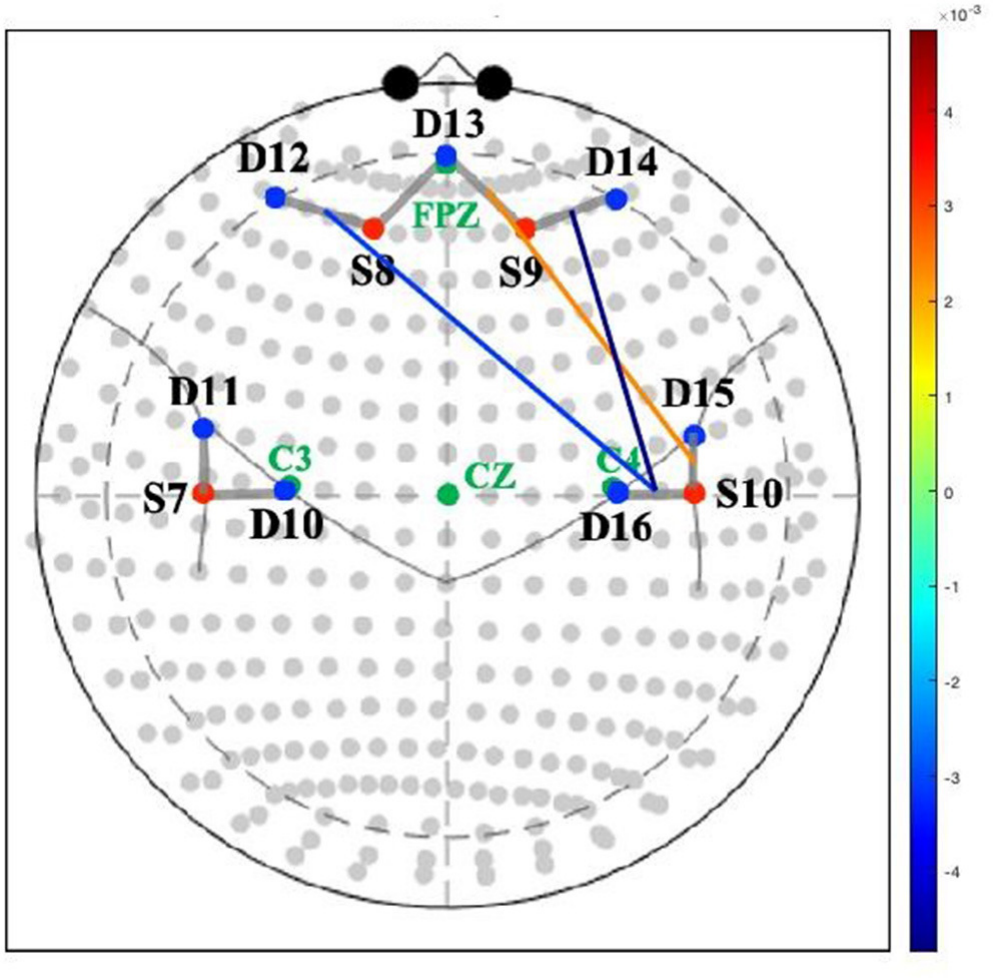
Association between maternal depressive symptoms and neural connectivity during mother-infant play. Colored lines represent magnitude (*t*-values) of connectivity between channels. S, source; D, detector.

Optical imaging was performed using a continuous-wave CW6 fNIRS system (TechEn, Milford, MA) at sampling rate of 20 Hz. The data were measured simultaneously at two wavelengths (690 nm and 830 nm). Light intensity was automatically adjusted by the system to provide optimal gain. A total of 8 channels were measured from 4 sources and 7 detectors. The distance between source and detector was 3 cm. Sensors were mounted on a neoprene cap sized based on head circumference. The probe extended over the inferior frontal gyrus to the parietal regions (see Figure 1). For each mother, the fNIRS cap was positioned according to the international 10–20 coordinate system with the center of the lower edge of the probe (detector 13) aligned with FpZ. All infants tolerated mothers' cap placement well.

### Preprocessing

The NIRS data were analyzed in Matlab™ (Math-works, Natick, MA) 2021a as part of an open-source AnalyzIR toolbox (37). Raw fNIRS signals were first resampled to 4 Hz and converted to changes in optical density. Then, the measured intensity data of the two wavelengths were converted to relative oxy- and deoxy-hemoglobin concentration changes using the modified Beer-Lambert law (38). First-level analyses used an autoregressive general linear model. This previously validated model has been demonstrated to show better sensitivity-specificity characteristics and statistically addresses both increased false-discovery rates introduced by serially correlated noise due to physiology in NIRS and outliers related to motion (37, 39).

### Analytic Plan

First, normality of variables was assessed by visual examination of probability plots. No serious departures from normality were detected. Next, to better characterize our sample, we ran a series of *t*-tests and correlations with demographic variables and primary variables of interest in SPSS. Lastly, for our primary hypothesis, we ran a general linear model using the AnalyzIR toolbox (37) in Matlab™ in which maternal depressive symptoms on the CES-D were evaluated as a predictor of maternal brain connectivity during the mother-infant interaction. Maternal brain connectivity was computed as the correlation coefficient between two of the eight channels. We included maternal sociodemographic risk as a covariate of no interest in the model. We then extracted estimates from the three significant channels at *p* < 0.05 to evaluate concurrent associations with maternal sensitivity as coded from the mother-infant free play task.

## Results

### Descriptive Statistics and Intercorrelations

There were no differences between depressed and healthy mothers in terms of child age, child gender, mother age, mother sociodemographic risk, or maternal sensitivity during the interaction (see Table 1). As expected, depressed mothers had higher CES-D scores compared to healthy mothers at 12 months postpartum (*M*_DEPRESSED_ = 22.64, *M*_HEALTHY_ = 3.17, *t* = −7.0*7, p* < 0.001). There was no significant association between maternal sensitivity and depressive symptoms on the CES-D, although the association was moderate in size (*r* = −0.31, *p* = 0.15).

**Table 1:** Descriptive statistics.

	**Total sample Ratio (%)**	**Healthy (*n* = 12) Ratio (%)**	**Depressed (*n* = 11) Ratio (%)**	**χ^**2**^, *p***
Sociodemographic risk:no risk ratio	8:15 (35% risk)	3:9 (25% risk)	5:6 (45% risk)	*X*^2^ = 1.06, *p* = 0.28
Child male:female ratio	14:9 (61% male)	6:6 (50% male)	8:3 (80% male)	*X*^2^ = 1.26, *p* = 0.40
	**Total sample ***M*** **(*****SD***)**	**Healthy (*****n*** **=** **12) ***M*** **(*****SD***)**	**Depressed (*****n*** **=** **11) ***M*** **(*****SD***)**	***t, p***
Child age in months	12.04 (1.40)	11.67 (1.37)	12.45 (1.37)	*t* = −1.38, *p* = 0.18
Mother age in years	31.41 (3.57)	32.33 (2.81)	30.30 (4.19)	*t* = 1.36, *p* = 0.19
Mother CES-D	12.48 (11.85)	3.17 (3.76)	22.64 (8.71)	*t* = −7.07, *p* < 0.001
Maternal sensitivity	4.23 (0.55)	4.38 (0.34)	4.06 (0.69)	*t* = 1.39, *p* = 0.18

### Association Between Depression and Neural Connectivity

Greater depression severity at 12-months postpartum was related to lower connectivity between the right TPJ and both the right lateral prefrontal cortex (*t* = −3.51, *p* < 0.01) and the left lateral prefrontal cortex (*t* = −2.56, *p* < 0.03) during the mother-infant book interaction. Greater depression severity was also related to greater connectivity between the right TPJ and anterior medial prefrontal cortex (*t* = 2.10, *p* < 0.05) during the mother-infant book interaction (see Table 2 and Figure 1).

**Table 2:** Maternal depressive symptoms on neural connectivity.

	**Origin number**	**Source region**	**Destination number**	**Detector region**	***t***	***p***
1	8, 12	Left Lateral PFC	10, 16	Right TPJ	−2.56	0.018
2	9,14	Right Lateral PFC	10, 16	Right TPJ	−3.51	0.002
3	9,13	Anterior Medial PFC	10, 15	Right TPJ	2.10	0.048

*Values presented are from oxyhemoglobin (HbO). No findings from deoxyhemoglobin (HbR) were significant*.

We explored associations among extracted correlation coefficients for our three significant channels and maternal sensitivity from the mother-infant free play task (see Table 3). Contrary to expectations, maternal sensitivity was not significantly correlated with neural connectivity from any of our three identified channels (*p*s = 0.16–0.91), although the association between maternal sensitivity and neural connectivity between the right TPJ and right lateral PFC was small to moderate in size (*r* = 0.24).

**Table 3:** Associations among maternal sensitivity and extracted neural connectivity values.

**Variables**	**1**	**2**	**3**
Maternal sensitivity	–	–	–
Right TPJ–Left lPFC connectivity	0.02	–	–
Right TPJ–Right lPFC connectivity	0.24	0.77^**^	–
Right TPJ–Anterior medial PFC connectivity	0.10	0.44^*^	0.42^*^

*Only the association between Right TPJ-Left lPFC Connectivity and Right TPJ-Right lPFC Connectivity was significant after Bonferroni corrections (i.e., p < 0.008)*.

** *p < 0.01*,

* *p < 0.05*.

## Discussion

Our findings demonstrate that women's postpartum depressive symptoms are associated with altered connectivity between mentalizing regions and affective processing regions when engaging with her infant even 12 months post-delivery. Specifically, we found that higher levels of maternal depressive symptoms were associated with *lower* connectivity between the right TPJ and lateral prefrontal cortex, but *higher* connectivity between the right TPJ and anterior medial prefrontal cortex in mothers during mother-baby interactions. The right TPJ is implicated in mentalizing, the ability to understand the thoughts and feelings of others (24, 25). The lateral prefrontal cortex plays a role in affect expression and regulation (21). Our finding that postpartum depressive symptoms are associated with lower connectivity between the right TPJ and the lateral PFC may imply that, in the context of depression, postpartum mothers' mentalizing about her infants' thoughts and feelings may be related to lower ability to express and regulate her own emotions.

On the other hand, postpartum mothers with greater depressive symptoms may link perceptions of their infant's thoughts and feelings with greater activity in regions implicated in affectionate touch and bonding, given the anterior medial prefrontal cortex's putative role in these functions (23). This finding is intriguing as it may suggest that not all depression-related brain alterations may be indicative of caregiving disruptions. Combined, these findings may suggest that, in the context of considering her infant's thoughts and feelings, although mothers with depressive symptoms may have less ability to express and regulate their own emotions, they may allocate *more* resources to soothing and bonding with their own infants.

Indeed, some models have characterized postpartum depression as a period of extreme concern and worry about the infant's wellbeing (11), which may be related to neural and behavioral changes that occur during the transition to motherhood and are associated with heightened salience of infant cues (40, 41). Although this type of “caring concern” may facilitate the provision of warm and loving care to her infant, increasing levels of this type of caring concern during the postpartum transition to motherhood may be distressing to some mothers, especially if they have doubts about their ability to provide the care that their infant needs. It may be that mothers with greater postpartum depressive symptoms experience higher levels of caring concern combined with feelings of emotional dysregulation. In this case, postpartum mothers with depressive symptoms may prioritize their infants' needs above managing their own emotions. This may be good news for infant socio-emotional development at this stage, as the late postpartum period coincides with important changes in the development of infant's emotional self-regulatory skills (42). Notably, the infant is engaging in more purposeful and self-motivated regulatory behaviors (e.g., gaze aversion, thumb/finger sucking) and infant emotional self-regulation is largely being formed *via repeated* co-regulation with the infant's caregiver (43, 44). Thus, having a regulated and responsive caregiver is key to healthy infant socio-emotional development at this stage (43). However, despite this neural profile potentially being advantageous for infant development, it is unclear whether the combination of increased connectivity in regions that facilitate caring concern but decreased connectivity in regions that may aid in self-regulation may interfere with maternal ability to recover from her own depressive symptoms during the postpartum period.

It is important to note that many mothers with elevated postpartum depressive symptoms in our study also met criteria for one or more anxiety or trauma-related psychiatric disorders. Thus, our findings may not be specific to the experience of postpartum depression but may also be related to postpartum psychiatric disorders in general. Recent research has shown that postpartum anxiety is related to neurobiological disruptions, such increased activation in the amygdala and insula, and decreased activity in prefrontal regions including the lateral prefrontal cortex [see (45) for a review]. Further, postpartum anxiety has been associated with disruptions in caregiving, most notably intrusive thoughts about baby's wellbeing and over-stimulating interactions with one's infant (45, 46). Our findings that mothers with greater depressive severity showed greater connectivity between the TPJ and regions involved in affectionate touch, while also demonstrating lower connectivity with regions implicated in self-regulation fall in line with this prior work. More research will be needed to distinguish neurobiological disruptions specific to postpartum depression from postpartum anxiety and examine how these disruptions may be related to sensitive vs. intrusive parental care. We also acknowledge that four mothers were currently using psychotropic medications which may have tempered our findings, although prior work has demonstrated that brain activity assessed *via* NIRS did not change with antidepressant medication use [see (47)].

There are several limitations to our study that warrant discussion. First, although our sample was comparable to or larger than other NIRS studies (19, 48), a larger sample would have allowed for a case-control design that directly compared neural activity in women with clinical levels of PPD to psychiatrically healthy women. Future work with larger samples may provide more opportunity to evaluate brain-behavior associations. It should be noted that we did not find that neural connectivity or postpartum depressive symptoms were significantly associated with maternal sensitivity in this preliminary study, which may have been due to reduced power to find effects given the magnitude of the correlations (and their small to moderate effect sizes). Further, a study that examined women who recovered from PPD by 12 months postpartum with those who still have clinical or residual levels of symptoms during this late postpartum period would be particularly illuminating. Follow up analyses that evaluated how these neural differences predict changes in mother and infant behavior over time would also provide greater context for these findings. Lastly, although our sample included women of various racial and ethnic backgrounds and levels of educational achievement, most of the mothers in our study were married, college-educated White women.

Nevertheless, our study has several strengths. First, we employed near infrared spectroscopy to evaluate mother neural response in regions implicated in affect and social cognition during an ecologically valid and personally relevant context, playing with one's own infant. Second, we examined how depressive symptoms during the late postpartum period, a forgotten time in which symptoms often persist and that coincides with the emergence of infant self-regulatory capacities, may be associated with altered maternal brain activity. Future work should explore how connectivity among socio-affective neural regions changes with improvements in mothers' depressive symptoms (e.g., with treatment) during the transition out of the postpartum period. Overall, our findings provide new knowledge that mothers with depressive symptoms in the late postpartum period may show alterations in mentalizing and affective processing regions, that may be indicative of their caregiving patterns. Although future work to replicate our findings is needed, our study underscores the importance of supporting maternal mental health, *past* the initial postpartum weeks and months, to both aid mothers' own emotional wellbeing and foster healthy infant development.

## Data Availability Statement

The original contributions presented in the study are included in the article, further inquiries can be directed to the corresponding author/s.

## Ethics Statement

The studies involving human participants were reviewed and approved by University of Pittsburgh Human Research Protections Office. Written informed consent to participate in this study was provided by the participants' legal guardian/next of kin.

## Author Contributions

JM formalized the research question, led study conceptualization and design, assisted with analytic strategy, and drafted the manuscript. HS conducted analyses and edited all sections of the manuscript. RF and KC led study management and edited all sections of the manuscript. EF and AH contributed to study conceptualization and design and edited all sections of the manuscript. TH contributed to study conceptualization and design and led data analytic strategy. All authors contributed to the article and approved the submitted version.

## Funding

This research was supported by R01 MH113777 from the National Institutes of Health to JM.

## Conflict of Interest

The authors declare that the research was conducted in the absence of any commercial or financial relationships that could be construed as a potential conflict of interest.

## Publisher's Note

All claims expressed in this article are solely those of the authors and do not necessarily represent those of their affiliated organizations, or those of the publisher, the editors and the reviewers. Any product that may be evaluated in this article, or claim that may be made by its manufacturer, is not guaranteed or endorsed by the publisher.
